# Evidence of large systematic differences between countries in assigning ischaemic heart disease deaths to myocardial infarction: the contrasting examples of Russia and Norway

**DOI:** 10.1093/ije/dyab188

**Published:** 2021-09-11

**Authors:** Sergey Timonin, Vladimir M Shkolnikov, Evgeny Andreev, Per Magnus, David A Leon

**Affiliations:** 1 International Laboratory for Population and Health, National Research University Higher School of Economics, Moscow, Russia; 2 Laboratory of Demographic Data, Max Planck Institute for Demographic Research, Rostock, Germany; 3 Norwegian Institute of Public Health, Oslo, Norway; 4 Department of Non-communicable Disease Epidemiology, London School of Hygiene and Tropical Medicine, London, UK; 5 Department of Community Medicine, UiT The Arctic University of Norway, Tromsø, Norway

**Keywords:** Myocardial infarction, ischaemic heart disease, validity of causes of death, autopsies, place of death, Russia, Norway

## Abstract

**Background:**

There is considerable variation in mortality rates from myocardial infarction (MI) across high-income countries, some of which may be artefactual.

**Methods:**

Time trends in mortality rates from ischaemic heart disease (IHD) and MI were analysed for a set of high-income countries from the end of the 1970s. Using individual-level mortality data from Russia (2005–2017) and Norway (2005–2016), we investigated factors associated with the proportion of total IHD deaths certified as due to MI.

**Results:**

In most countries, MI mortality rates have dramatically declined from the 1970s. However, the share of MI in total IHD deaths varies substantially across countries. In Russia, only 12% of IHD deaths had MI assigned as the underlying cause vs 63% in Norway. IHD deaths occurring outside of hospital without autopsy were far less likely to be assigned as MI in Russia (2%) than in Norway (59%).

**Conclusions:**

Although established international criteria for MI require specific clinical or post-mortem evidence, it appears that certifying specialists in different countries may interpret these criteria differently. At one extreme, Russian doctors may only assign MI as a cause of death when there is specific pathophysiological evidence. At the other extreme, their counterparts in Norway may be willing to specify MI as the cause even when this evidence is not available. Internationally established criteria for MI diagnosis are challenging to apply for out-of-hospital deaths. Differences between countries in how certifiers interpret these criteria may account for at least some of the international variation in MI mortality rates.

Key Messages

- There are considerable international differences in population-level rates of mortality from myocardial infarction (MI). Some of this variation may be explained by differences between countries in the minimal criteria required by certifying doctors to specify MI as the underlying cause of death.
- At one extreme, in Russia there is a tendency for certifiers to ascribe MI as the underlying cause only in contexts in which pathophysiological evidence is likely to be available, which in most cases will be if the death occurs in hospital and/or post-mortem autopsy is performed. In contrast at the other extreme, in Norway certifying doctors appear more willing to specify MI as the underlying cause even for deaths occurring at home in the absence of pathophysiological evidence. Further research is warranted looking in detail at other countries.
- Despite the considerable efforts of expert groups in developing formal criteria for determining whether a death could be classified as due to an MI, we have identified evidence of substantial differences in routine certification practice that mean that these data may not be meaningfully comparable across countries.

## Introduction

Understanding the nature of between- and within-country variation in mortality from cardiovascular diseases (CVDs) is crucial for promoting better cardiovascular health. Much of the between-country differences will be explained by differences in exposure to risk factors and medical care. However, as has been well documented, within the overall class of CVD deaths, differences in the certification and coding of deaths almost certainly make a contribution to international variation.^1–9^

Cardiovascular mortality rates in Russia have been among the highest in the world for many decades and remain so despite the declines that have occurred since the mid-2000s.^10^^,^^11^ The exceptionally high rates in Russia together with its large population mean that Russia makes a substantial regional contribution to CVD mortality. Within the World Health Organization (WHO) European region in 2019, Russia accounted for almost a quarter (23%) of all deaths assigned to ischaemic heart disease (IHD) as the underlying cause.^12^ However, there have been very few studies^2^^,^^9^ of the international comparability of cardiovascular mortality rates that have included Russia.

Myocardial infarction (MI) is an important component of IHD mortality, information on which is reported by high-income countries as part of their routine mortality statistics. These data are used by researchers and policy makers looking at within-country differences and trends,^13^ particularly in the context of the impact of hospital admission.^14^ A number of studies have looked at the within-country validity of MI as a cause of death. These have been reviewed by McCormick *et al*.,^15^ who concluded that researchers should avoid using vital-statistics data on deaths from MI if hospitalization data are not available to confirm the cause of death. However, of the studies reviewed, only five looked at routine cause-of-death data, and these only covered deaths in the period 1984–1993. However, little work has been done to consider the degree to which variation in MI mortality rates between high-income countries may be driven by artefacts of certification and coding.

The WHO and professional societies of cardiology have attempted to standardize the definition of MI, which has led to increasingly well-defined objectively measured clinical criteria.^16–21^ The most recent guidelines lay out specific, largely pathophysiological criteria for establishing a death from MI.^21^ How far these criteria have been adopted by certifying experts in different countries is unknown.

This study was originally motivated by our observation of an apparent paradox in patterns of mortality rates from IHD overall and MI in Russia. Although Russia has had one of the highest IHD mortality rates among industrialized countries, it has one of the lowest reported and stable rates of mortality from MI.^22^^,^^23^

In this paper, we report the results of an investigation of how far the low rates of mortality from MI in Russia compared with other countries could be explained by differences in how the certification of MI as the underlying cause may be influenced by the place of death and the likelihood of an autopsy being performed. Although cardiovascular epidemiologists are aware of the challenge of interpreting routine cause-of-death data,^3^^,^^4^^,^^6^^,^^7^^,^^9^^,^^15^ the potential role of artefact in explaining differences between countries in MI mortality within the larger group of all IHD deaths has not been previously investigated.

## Data and methods

We used routine mortality data for Russia, Norway and a number of other comparator countries representing different geopolitical regions (Australia, Czechia, Estonia, France, Japan, the UK and the USA) to explore the patterns and structure of IHD mortality between countries. We then examined how individual-level factors recorded on the death certificate influence whether an IHD death was classified as being due to MI in Russia compared with Norway.

### Population-level analysis

For countries except Russia, we used data on deaths by age, sex and cause from the WHO Mortality Database^24^ and population exposures from the Human Mortality Database.^25^ For Russia, death rates were obtained from the Russian Fertility and Mortality Database for the whole available period of 1965–2017.^26^^,^^27^ We divided all cardiovascular diseases into three groups: (i) MI (ICD-9 codes: 410; ICD-10 codes: I21, I22), (ii) the rest of the IHDs (ICD-9 codes: 411–414; ICD-10 codes: I20, I23–I25), (iii) the rest of the CVDs (ICD-9 codes: 390–409, 415–459; ICD-10 codes: I00–I19, I26–I99).

We used the European Population Standard (1976) to calculate age-standardized death rates for the adult population (ages 30+ years).^28^

### Individual-level analysis

Anonymized individual-level data on all IHD deaths in Russia (2005–2017) were obtained from the Russian State Statistical Service (Rosstat). Equivalent data for Norway (2005–2016) were provided by the Norwegian Institute of Public Health (NIPH). These two countries, which share a common border, report the lowest and highest proportion of MI deaths among all IHD deaths, respectively. Moreover, they have been the subject of international comparative studies looking at determinants of differences in CVD risk between them.^29–31^ Although we wanted to include other countries in this part of our analysis, attempts to obtain equivalent micro-level data for other countries were unsuccessful.

Multiple logistic regression was used to estimate the effect of individual-level factors on the odds of an IHD death being certified as being due to MI vs the rest of the IHDs. The dependent variables were sex, age (10-year age groups: 30–39, …, 80+), urban/rural residence, year of death, autopsy (yes/no) and place of death (hospital/elsewhere). The two latter variables are specified on death certificates and indicate the likelihood of pathophysiological evidence of an MI being available.

## Results

**Figure 1:**
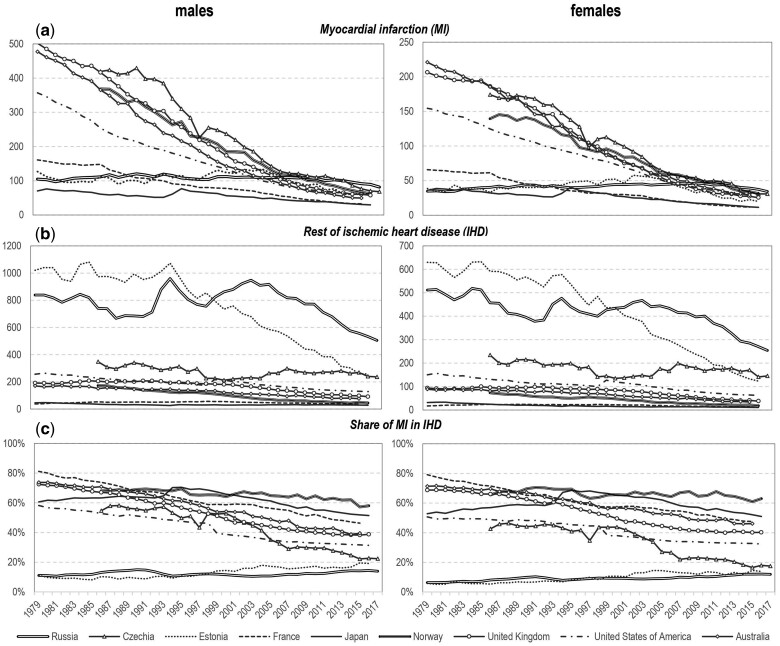
Age-standardized death rates (per 100 000) for (A) myocardial infarction (MI), (B) the rest of the ischaemic heart diseases (IHDs) and (C) the share of MI in IHD (in %) by sex and countries, since the introduction of ICD-9 Source: Russian Fertility and Mortality Database for Russia; World Health Organization (WHO) Mortality Database for reference countries.

Figure 1 shows time trends of mortality rates from MI, the rest of the IHDs and the rest of the CVDs by country. MI mortality rates in all countries in Figure 1 other than Russia and Estonia (another post-Soviet country) declined from the 1970s. Some of the falls were particularly steep, resulting in a convergence in MI rates between countries. In Russia, the rate of MI mortality was lower than in most of the comparator countries until the mid-2000s, but at a similar level to Estonia and Japan in contrast to the rest of IHDs, for which Russia had the highest rate throughout.

Although the rates for MI remained relatively stable and low in Russia, mortality rates from the rest of the IHDs and the rest of the CVDs have been subject to substantial fluctuations particularly among men (Supplementary Figure S1, available as Supplementary data at *IJE* online, for more details). In Russia in 2005, IHD mortality (excluding MI) entered a phase of sustained decline, but it was only in 2010 that mortality rates from MI began to decrease. These trends contrast sharply with those seen in all other countries studied (apart from Estonia), which did not show major fluctuations in mortality rates for the rest of the IHDs (Figure 1).

Unlike the absolute rates of MI mortality, the proportional contribution of MI to overall IHD mortality has not shown much convergence and still shows appreciable international variation, with Russia having the lowest and Norway having the highest proportion of IHD deaths accounted for by MI (Figure 1, lower panel). Of the other countries, Estonia exhibits trends that were similar to Russia, whereas Norway is at the opposite extreme, showing the highest and almost unchanged proportion of IHD deaths certified as being due to MI. Much of the decline in MI in Russia since 2010 is accounted for by a fall in rates for deaths occurring in hospital (27% and 30% for males and females, respectively) whereas death rates among those dying out of hospital have changed very little. In contrast, the substantial decline in mortality rates from the rest of the IHDs has been mostly driven by a fall in mortality among those who died at home (38% and 41% for males and females, respectively).

Using individual-level data, we turn to looking at the influence of the place of death and whether an autopsy was conducted on the probability of an IHD death being certified as being due to MI for Russia and Norway. Overall, 81% of IHD deaths occurred outside of hospital, the equivalent figure for Norway being 89%. Table 1 shows the number and proportion of overall IHD deaths accounted for by MI according to age, place of death and autopsy status in the two countries. In Russia, the highest proportion of IHD deaths assigned to MI are among those dying in hospital and who had an autopsy (44%) whereas those who died outside of hospital and did not have an autopsy had the lowest proportion assigned to MI (2%). Norway showed an even higher proportion of IHD deaths assigned to MI among those who died in hospital and had an autopsy (60%). However, in Norway, there was also a high proportion of MI deaths among those dying out of hospital without an autopsy (59%). The contrast between the two countries for deaths outside of hospital without an autopsy is particularly dramatic among those aged 80+ years. At this older age, MI constituted <1% of IHD in Russia and 56% of IHD in Norway (Table 1).

**Table:** Table **1** Distribution of myocardial infarction (MI) and the rest of the ischaemic heart disease (IHD) deaths by age, place of death and autopsy in Russia (2005–2017) and Norway (2005–2016)

	Deaths in hospital						Deaths out of hospital						Total deaths		
Ages (years)	with autopsy			without autopsy			with autopsy			without autopsy					
	MI	Rest of IHD	Share of MI	MI	Rest of IHD	Share of MI	MI	Rest of IHD	Share of MI	MI	Rest of IHD	Share of MI	MI	Rest of IHD	Share of MI
	Russia^a^														
30–49	17	20	46.4%	3	4	43.4%	26	226	10.3%	3	27	9.4%	49	277	15.0%
50–59	52	55	48.3%	12	19	39.8%	56	461	10.8%	7	123	5.5%	127	658	16.2%
60–69	90	100	47.3%	27	50	34.9%	62	502	11.0%	11	400	2.6%	189	1052	15.3%
70–79	133	161	45.3%	53	115	31.6%	62	527	10.5%	17	1158	1.4%	265	1962	11.9%
80+	91	154	37.0%	39	116	25.0%	46	563	7.6%	11	1412	0.8%	187	2245	7.7%
All ages	383	490	43.8%	134	304	30.6%	252	2279	9.9%	49	3119	1.6%	817	6192	11.7%
	Norway														
30–49	107	57	65.2%	198	244	44.8%	165	27	85.9%	259	48	84.4%	729	376	66.0%
50–59	217	107	67.0%	334	500	40.0%	434	116	78.9%	793	241	76.7%	1778	964	64.8%
60–69	390	230	62.9%	424	729	36.8%	1118	426	72.4%	1924	732	72.4%	3856	2117	64.6%
70–79	584	394	59.7%	235	410	36.4%	2854	1176	70.8%	3462	1860	65.1%	7135	3840	65.0%
80+	661	506	56.6%	84	212	28.4%	10 231	3139	76.5%	13 083	10 500	55.5%	24 059	14 357	62.6%
All ages	1959	1294	60.2%	1275	2095	37.8%	14 802	4884	75.2%	19 521	13 381	59.3%	37 557	21 654	63.4%

Source: Estimated from micro-level mortality data provided by Rosstat and the Norwegian Institute of Public Health (NIPH) upon request.

a The number of deaths in Russia are in thousands.

Table 2 shows the odds ratios (ORs) for having MI specified as the underlying cause among all IHD deaths according to the place of death (hospital/elsewhere) and whether an autopsy of any type was conducted (yes/no). In Russia, the OR for having MI as an underlying cause among all IHD deaths was 46 for deaths in hospital that were autopsied relative to those occurring outside of hospital without an autopsy. There was a progressive decline in OR across the other categories. However, in Norway, the place of death and having an autopsy showed far weaker associations with whether an IHD death was assigned to MI or not. More detailed results from this analysis are presented in Supplementary Table S1 (available as Supplementary data at *IJE* online).

**Table 2:** Adjusted^a^ odds ratios (95% CIs) for having myocardial infarction (MI) specified as the underlying cause among all deaths from ischaemic heart disease (IHD) in Russia and Norway

	Russia (2005–2017)			Norway (2005–2016)		
	Odds ratio (95% CI)	Number of deaths		Odds ratio (95% CI)	Number of deaths	
		MI	Rest of IHD		MI	Rest of IHD
Deaths in hospital, autopsy	46.49 (46.02–46.96)	382 768	490 767	0.90 (0.84–0.97)	1959	1294
Deaths in hospital, no autopsy	28.84 (26.54–27.14)	133 969	303 803	2.08 (2.00–2.17)	14 802	4884
Deaths out of hospital, autopsy	6.11 (6.05–6.17)	251 619	2 281 886	0.30 (0.27–0.32)	1275	2095
Deaths out of hospital, no autopsy	1.00 [ref]	49 173	3 119 228	1.00 [ref]	19 521	13 381

Source: Estimated from anonymized individual-level data provided by Rosstat and the Norwegian Institute of Public Health (NIPH) upon request.

a Adjusted for sex, age, year and place of residence. CI, confidence interval.

One feature of IHD deaths in Russia compared with many other countries is that a much greater proportion of them are assigned to I25.0 and I25.1. In order to try and make the category of non-IHD deaths more comparable with those in Norway, we repeated the analyses presented in Table 2 having excluded I25.0 and I25.1. As shown in Supplementary Table S2 (available as Supplementary data at *IJE* online), although the Russian ORs were slightly attenuated, very strong associations with place of death and autopsy remained.

**Figure 2:**
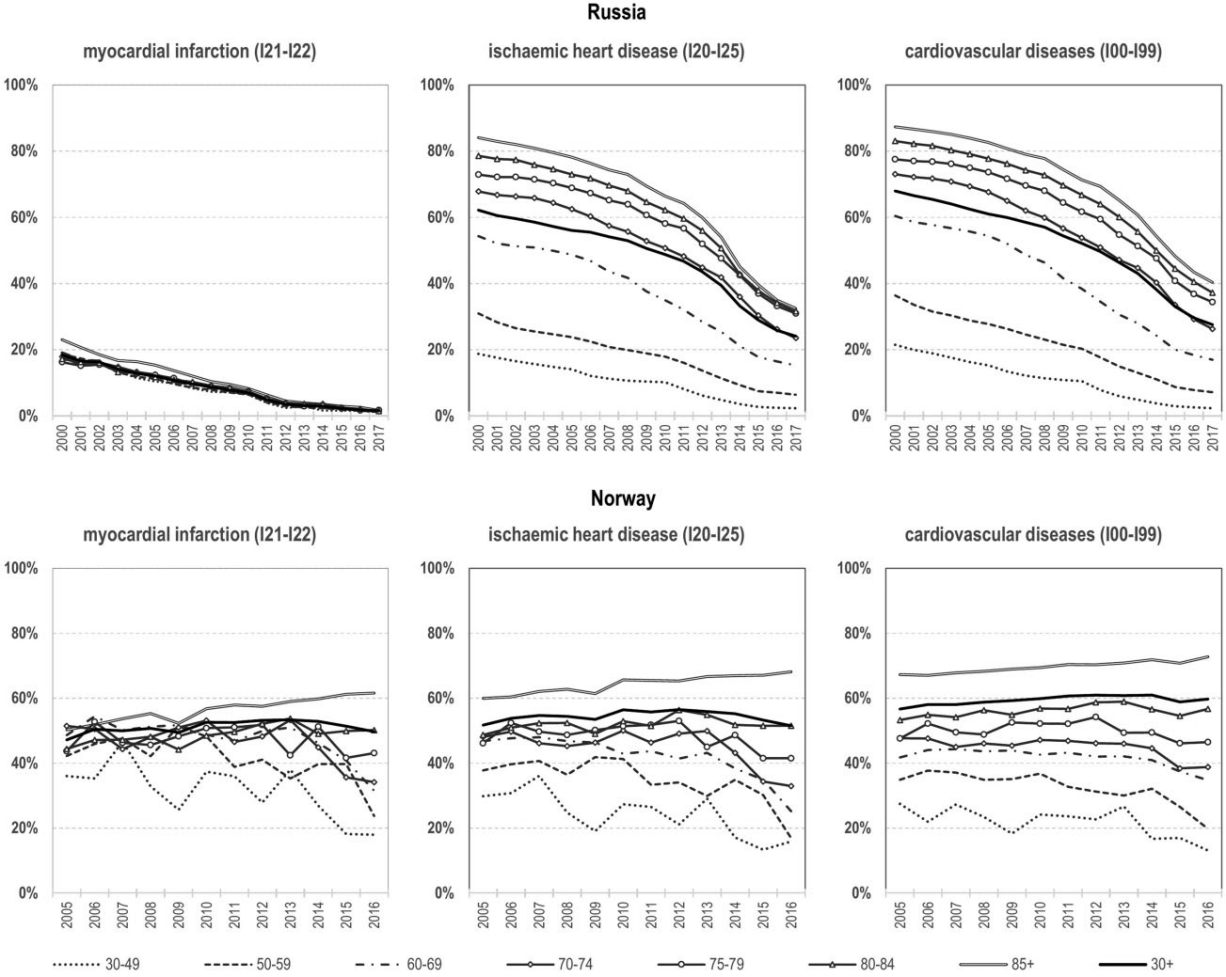
Age-specific percentages of myocardial infarction (MI), ischaemic heart disease (IHD) and cardiovascular disease (CVD) deaths that occurred outside of hospital without an autopsy, by year of death, Russia(2000–2017) and Norway (2005–2016) Source: Calculated from micro-level mortality data provided by Rosstat and the Norwegian Institute of Public Health (NIPH) upon request.

In the final part of our analyses, we considered how far changes in where deaths occurred in Russia and Norway might explain patterns of MI mortality. In Russia since 2000, there has been a steep decline in the percentage of deaths that occurred outside of hospital and did not have an autopsy. This is apparent for all three classes of CVD (Figure 2, upper panel). The decline is steepest for IHD in particular at ages 70+ years and in the most recent years. MI showed the lowest percentage of deaths occurring outside of hospital without an autopsy: 20% in 2000 falling to 2% in 2017. Put the other way around, the proportion of CVD deaths occurring in hospital or with an autopsy has increased substantially over time in Russia, especially at older ages. In the case of MI, the share of such deaths constituted 98% in 2017 with almost no variation by age and sex. In Norway, on the contrary, the share of deaths that occurred outside of hospital with no autopsy have been relatively stable over time and across CVD categories (Figure 2, lower panel). On average, 51%, 54% and 59% of MI, IHD and CVD deaths, respectively, occurred in out-of-hospital settings without subsequent post-mortem examination in Norway in 2005–2016.

## Discussion

Compared with Norway, and most other countries that we have examined, MI mortality rates in Russia have been low and stable. In contrast, IHD mortality rates in Russia have been the highest. In Russia (2005–2017), only 12% of IHD deaths had MI assigned as the underlying cause. This was the lowest proportion of all countries, with Norway having the highest at 63% (2005–2016). Most importantly, we have found that in Russia, the place of death and whether an autopsy was conducted were strongly related to the share of IHD deaths accounted for by MI, whereas in Norway, these factors had a far weaker influence. IHD deaths occurring outside of hospital without autopsy were far less likely to be assigned to MI in Russia (2%) than in Norway (59%). We suggest that these large contrasts between Russia and Norway may be best explained by differences in how certifying doctors apply the international criteria to certify a presumptive death from IHD as being due to MI.

Up until the 2000s, the definition of MI was based on the so-called epidemiological approach (primarily electrocardiography-based).^21^ MI would be diagnosed if the following clinical features and their combinations were present: corresponding ECG changes, typical symptoms of acute ischaemia and elevated enzymes. Added to this, a diagnosis of a fatal case in the absence of these clinical characteristics observed in life required the naked-eye appearance of a fresh infarction and/or recent coronary occlusion found at autopsy.^16^ The most recent guidelines specify that the fundamental criteria for establishing a death from MI is the detection of abnormal cardiac biomarkers (e.g. troponin) in the setting of evidence of acute myocardial ischaemia (suggestive symptoms or new ischaemic ECG changes) and/or post-mortem evidence of a fresh thrombus occluding a coronary artery with recent myocardial damage.^21^ Previous work has shown that changes in these sorts of criteria can have a substantial effect on the rates of incident and prevalent MI.^32^

We suggest that, in Russia, doctors may be particularly reluctant to certify a death as being due to acute MI without there being the sort of specific pathophysiological evidence discussed in the previous paragraph. Such evidence will not usually be available for sudden deaths occurring outside of hospital, particularly those that are not subject to autopsy. In contrast, it appears that doctors in Norway may be more willing to certify deaths as due to MI based only on reported symptoms and prior clinical history. What is notable is that Estonia, which was part of the Soviet Union until the early 1990s, also has a particularly low proportion of IHD deaths classified as due to MI even in recent years. This may suggest that any distinctive certification practices established in the Soviet period have persisted.

The impact of the correct but conservative approach to certifying MI deaths in Russia will inevitably lead to an underestimate of the true rate of MI mortality overall, as only 20% of IHD deaths in Russia occur in hospital; the vast majority happen at home. In contrast, in Norway, those dying at home are only a little less likely to have MI assigned as the underlying cause of death compared with those dying in hospital. This could reflect the fact that Norwegian certifiers are willing to certify a death as such even in the absence of direct evidence such as ECG and serum troponin levels and/or the presence of a thrombus or recent myocardial injury at an autopsy. Notably, Norway has one of the highest proportions of IHD deaths certified as being due to MI of any of the comparator countries that we have looked at.

One of the unusual features of IHD mortality in Russia is that it has shown very sharp fluctuations in mortality since the mid-1980s. This has been attributed to simultaneous fluctuations in harmful alcohol consumption.^33^ However, as noted originally by Zaridze *et al*.,^23^ mortality rates from MI in Russia have shown almost no association with harmful alcohol consumption. Our conclusion that the true-positive rate of MI deaths in Russia is likely to be high is consistent with the notion that although classic atherosclerotic-related MI may not be related to alcohol, there is an important fraction of deaths attributed to non-MI IHD that are associated with alcohol. As we have already noted, compared with other countries, Russia uses ICD codes I25.0 and I25.1 frequently as an underlying cause of death, despite the view that they may be garbage causes that should not be used on death certificates.^34^ However, our conclusions in this paper concerning the very strong association of the place of death and autopsy with an IHD death being assigned to MI was found even when deaths from I25.0 and I25.1 were excluded from our analysis of individual-level data (Supplementary Table S2, available as Supplementary data at *IJE* online). This suggests that our results are not driven by the inflation of non-IHD deaths with the opaque category of deaths coded to I25.0 and I25.1.

In summary, our analysis suggests that Russia is an example of a country where those who certify cause of death are conservative in their approach, and may adhere to the formal criteria for MI more strongly than in many other countries. In contrast, Norway may have a more relaxed approach to applying these criteria, which would explain the very high proportion of deaths assigned to MI and the very weak effect of the place of death or having an autopsy on the likelihood of certifying an MI. In other words, Russia is likely to have a low percentage of false-positive cases but could have a higher percentage of false-negative cases (deaths out of hospital). Norway might have a high percentage of false-positive cases (deaths at home) but a lower percentage of false-negative cases.

Our analyses provide important insights into the international comparability of MI mortality rates. At the extremes examined (Russia vs Norway), it appears that differences in rates are likely to be strongly influenced by differences in the minimal indications required for certifying a death as being due to an MI. Further work should be done in Russia and Norway to further test our conclusions and to investigate this issue in other countries through parallel analyses of micro-level data taking account of the place of death and whether an autopsy was conducted. Analysis of MI mortality trends within individual countries should take account of any changes that there may have been in the proportion of people with a suspected MI who are hospitalized, as this may impact certification rates.

More broadly, our analyses lead us to question how far the considerable efforts of expert groups in developing formal criteria for determining whether a death could be determined as due to an MI have so far resulted in data that are meaningfully comparable across countries, consistently with previous work questioning the usefulness and validity of MI determined on the basis of death-certificate mentions alone. Given that, in all countries, only a proportion of deaths occur in hospital, the scope for being able to more accurately identify all deaths as being due to an MI appears to be limited.

## Supplementary data

Supplementary data are available at *IJE* online.

## Ethics approval

Ethics approval is not needed as the study uses either publicly available macro-level (aggregated) mortality data or anonymized death records routinely collected by national statistical offices.

## Funding

The paper was in part prepared within the framework of the HSE University Basic Research Program. This work was also partly funded through the International Project on Cardiovascular Disease in Russia (IPCDR) supported by a Wellcome Trust Strategic Award [100217/Z/12], Norwegian Ministry of Health, Norwegian Institute of Public Health, UiT The Arctic University of Norway.

## Data availability

The aggregated mortality data were derived from sources in the public domain: WHO Mortality Database (https://www.who.int/data/data-collection-tools/who-mortality-database), the Human Mortality Database (https://www.mortality.org) and the Russian Fertility and Mortality Database (http://demogr.nes.ru/en/demogr_indicat). The individual-level data (anonymized death records) were provided by Rosstat for Russia and by the Norwegian Institute of Public Health (NIPH) on request.

## Supplementary Material

dyab188_Supplementary_Data
