# Supplementary material for: Evidence of large systematic differences between countries in assigning ischaemic heart disease deaths to myocardial infarction: the contrasting examples of Russia and Norway
Source: Int J Epidemiol. 2021 Sep 11;50(6):2082–90. doi: 10.1093/ije/dyab188 (PMC8743129; doi:10.1093/ije/dyab188)
Supplement: dyab188_Supplementary_Data [file dyab188_supplementary_data.docx]

**Supplementary tables and figures**


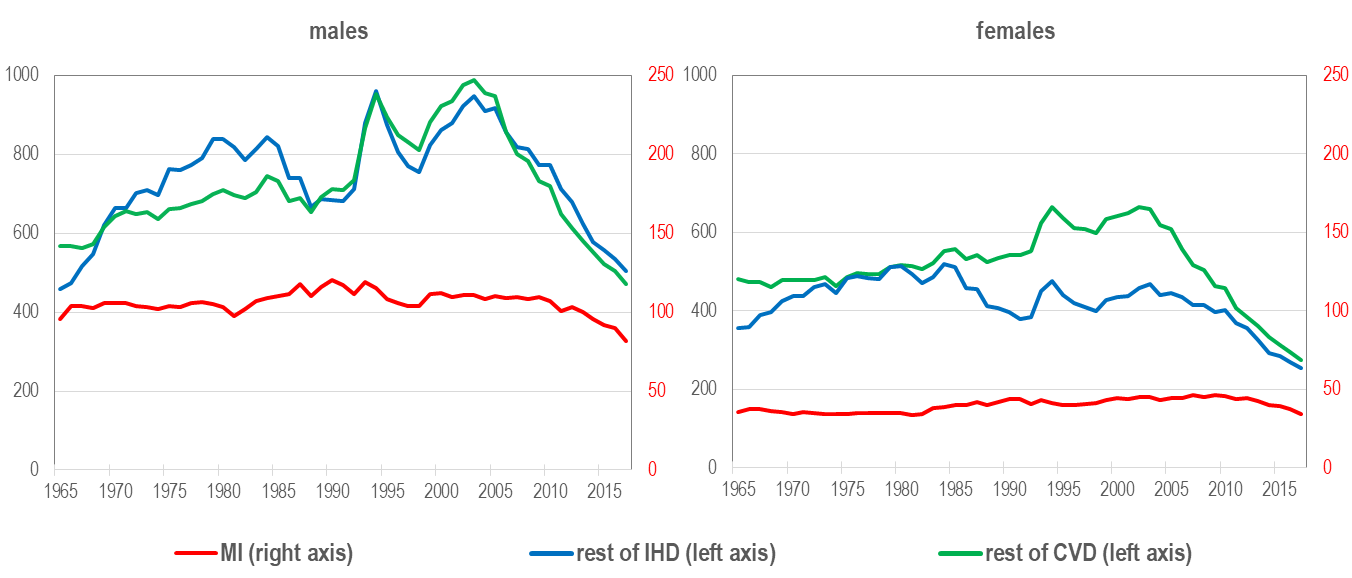


**Figure S1. Age-standardised death rates from myocardial infarction (MI), rest of ischaemic heart disease (IHD) and rest of cardiovascular disease (CVD) in Russia, per 100 000, by sex, 1965-2017.**

*Source:* Russian Fertility and Mortality Database^26^

**Table S1. Mutually adjusted odds ratios (95% CIs) for various factors for having myocardial infarction (MI) specified as the underlying cause among all deaths from ischaemic heart disease (IHD) in Russia (2005-2017) and Norway (2005-2016)**

| **Factor** | **Variable** | **Russia** | | | **Norway** | | |
| --- | --- | --- | --- | --- | --- | --- | --- |
|  |  | **ORs**  **(95% CI)** | **Number of deaths** | | **ORs (95% CI)** | **Number of deaths** | |
|  |  |  | **MI** | **rest of IHD** |  | **MI** | **rest of IHD** |
| **Age groups** | 30-39 | 0.81 (0.79 - 0.83) | 8 820 | 59 279 | 1.25 (0.91 - 1.72) | 123 | 78 |
|  | 40-49 | 0.99 (0.98 - 1.00) | 42 045 | 229 647 | 1.30 (1.10 - 1.52) | 666 | 349 |
|  | *50-59* | *1.00 [ref]* | *131 309* | *681 934* | *1.00 [ref]* | *1879* | *1057* |
|  | 60-69 | 0.93 (0.93 - 0.94) | 194 431 | 1 079 555 | 0.79 (0.72 - 0.87) | 3996 | 2244 |
|  | 70-79 | 0.79 (0.78 - 0.79) | 271 962 | 2 006 873 | 0.61 (0.55 - 0.67) | 7294 | 3949 |
|  | 80+ | 0.55 (0.54 - 0.55) | 190 605 | 2 287 145 | 0.50 (0.46 - 0.55) | 24353 | 14550 |
| **Sex** | *Females* | *1.00 [ref]* | *386 102* | *3 367 494* | *1.00 [ref]* | *17 992* | *10 183* |
|  | Males | 1.10 (1.09 - 1.10) | 453 278 | 2 980 567 | 0.91 (0.88 - 0.94) | 20 319 | 12 044 |
| **Year of death** | *2005* | *1.00 [ref]* | *63 262* | *560 650* | *1.00 [ref]* | *3936* | *2140* |
|  | 2006 | 1.03 (1.02-1.05) | 63 969 | 542 219 | 0.95 (0.88 - 1.03) | 3729 | 2136 |
|  | 2007 | 1.07 (1.05-1.08) | 65 618 | 527 323 | 1.02 (0.95 - 1.10) | 3780 | 2073 |
|  | 2008 | 1.04 (1.02-1.05) | 65 384 | 532 961 | 1.08 (1.00 - 1.17) | 3703 | 1927 |
|  | 2009 | 1.05 (1.04-1.07) | 67 246 | 516 732 | 0.93 (0.86 - 1.00) | 3381 | 2013 |
|  | 2010 | 0.98 (0.97-1.00) | 66 709 | 530 131 | 1.01 (0.94 - 1.10) | 3356 | 1876 |
|  | 2011 | 0.99 (0.97-1.00) | 64 894 | 502 200 | 1.04 (0.96 - 1.13) | 3218 | 1757 |
|  | 2012 | 0.97 (0.96 - 0.99) | 67 289 | 494 631 | 1.02 (0.94 - 1.10) | 3095 | 1773 |
|  | 2013 | 0.96 (0.95 - 0.98) | 66 166 | 462 722 | 0.96 (0.88 - 1.04) | 2796 | 1693 |
|  | 2014 | 0.93 (0.91 - 0.94) | 63 733 | 427 462 | 0.95 (0.87 - 1.03) | 2543 | 1564 |
|  | 2015 | 0.87 (0.86 - 0.88) | 63 637 | 430 223 | 0.87 (0.80 - 0.94) | 2470 | 1748 |
|  | 2016 | 0.82 (0.81 - 0.83) | 62 840 | 418 239 | 0.92 (0.85 - 1.01) | 2304 | 1527 |
|  | 2017 | 0.76 (0.75 - 0.77) | 58 633 | 402 568 | - | | |
| **Place of residence** | *Rural* | *1.00 [ref]* | *159 556* | *1 999 808* | - | | |
|  | Urban | 1.33 (1.32 - 1.34) | 679 824 | 4 348 253 |  |  |  |
| **Place of death** | In hospital | 10.43  (10.38 - 10.49) | 516 737 | 794 570 | 2.06 (1.98 - 2.13) | 16 761 | 6 178 |
|  | *Elsewhere* | *1.00 [ref]* | *300 792* | *5 401 114* | *1.00 [ref]* | *20 796* | *15 476* |
| **Autopsy performed** | *No* | *1.00 [ref]* | *200 495* | *3 542 338* | *1.00 [ref]* | *34 854* | *18 540* |
|  | Yes, pathology | 2.84 (2.82 - 2.86) | 638 885 | 2 805 723 | 0.48 (0.45 - 0.52) | 2 509 | 1 742 |
|  | Yes, forensic |  |  |  | 0.21 (0.19 – 0.23) | 945 | 1 944 |

*Source: estimated from anonymized individual-level data provided by Rosstat and Norwegian Institute of Public Health (NIPH) on request*

**Table S2. Adjusted* odds ratios (95% CIs) for having MI specified as the underlying cause among all deaths from IHD and among IHD deaths excluding ICD codes I25.0 and I25.1 in Russia (2005-17)**

|  | **Main estimations** | | | **Sensitivity analysis** | | |
| --- | --- | --- | --- | --- | --- | --- |
|  | **Odds Ratio**  **(95% CI)** | **Number of deaths** | | **Odds Ratio**  **(95% CI)** | **Number of deaths** | |
|  |  | **MI** | **rest of IHD** |  | **MI** | **rest of IHD (excluding I25.0 and I25.1)** |
| Deaths in hospital, autopsy | 46.49  (46.02 - 46.96) | 382 768 | 490 767 | 29.28  (28.97 - 29.59) | 382 768 | 267 860 |
| Deaths in hospital,  no autopsy | 28.84  (26.54 - 27.14) | 133 969 | 303 803 | 18.60  (18.38 - 18.82) | 133 969 | 150 304 |
| Deaths out of hospital, autopsy | 6.11  (6.05 - 6.17) | 251 619 | 2 281 886 | 4.30  (4.26 - 4.35) | 251 619 | 1 188 634 |
| Deaths out of hospital,  no autopsy | *1.00 [ref]* | 49 173 | 3 119 228 | *1.00 [ref]* | 49 173 | 1 058 577 |

*Source: estimated from anonymized individual-level data provided by Rosstat on request*

*Note: * Adjusted for sex, age, year, place of residence*
